# Supplementary material for: Risk and Clinical Significance of Idiopathic Preterm Birth in Microvillus Inclusion Disease
Source: J Clin Med. 2021 Aug 31;10(17):3935. doi: 10.3390/jcm10173935 (PMC8432107; doi:10.3390/jcm10173935)
Supplement: Supplementary file 1 [file jcm-10-03935-s001.zip › jcm-1303346-supplementary.pdf]

Supplementary Table 1. List of all cases of MVID with reported gestational age and clinical data. For legend see box at the end of the table.

PMID	GA (week	spont birt	seks (1: m	BW (g)	BW%	PH (1: yes	antenatal	onset (d)	stool (mg/	LD (1: yes,	RD (1: yes	TPN (1: ye	TPN wean	dead (1: y	last follow	Mutation	MYO5B mutation (f, m)	
28842815	38	1	1	3500	78.21	nr	2	3	nr	1	nr	1	2	1	1 mo	nr		
28842815	34	1	2	2000	40.02	nr	2	5	nr		1	1	2	1	1 mo	no *MYO5B*		
29546954	36	2	1	nr		2	2	10	150		1	1	2	2	36 mo	nr		
23525737	AT	nr	1	nr		1	2	6	150	1	nr	1	2	2	168 mo	nr		
23354788	35	2	2	2330	50.15	1	1	1	nr		1	1	2	1	7 mo	*MYO5B*	nr	nr
23226823	AT	1	2	nr		2	1	3	nr	1	nr	1	2	1	1 mo	nr		
22318102	35	1	2	2320	49.13	1	1	3	nr		nr	1	2	1	1 mo	nr		
22197941	37	1	2	2900	59.46	1	2	1	100	nr	nr	1	2	2	0 mo			
22152886	35	1	1	nr		nr	nr	4	nr	1	nr	1	2	2	41 mo			
21968248	AT	1	1	2734		2	2	3	175	1	nr	1	2	2	3 mo	nr		
21299349	35	1	1	nr		nr	2	3	nr		1	1	2	1	2 mo	nr		
18277898	34	1	1	2450	73.00	2	2	nr	nr		2	1	2	2	12 d			
17418172	31	1	1	1710	68.85	nr	nr	3	100	1	nr	1	2	2	12 d			
15456973	37	1	2	2530	24.52	nr	2	1	200	nr	nr	1	2	1	1.5 mo			
11783915	AT	1	1	nr		nr	2	6	115	nr	nr	1	2	2	5 mo	*MYO5B*	p.P660L	p.P660L
11414303	36	1	1	2700	50.98	2	2	2	200	nr	nr	1	2	2	4 mo	pre 2008		
11251929	35	2	1	3720	99.46	1	1	nr	300	nr	nr	1	2	2	3 d	pre 2008		
11173328	36	1	1	2740	54.78	2	2	3	nr	nr	nr	1	2	1	6 mo	pre 2008		
10941974	AT	1	2	3510		nr	2	11	nr	nr	nr	1	1	2	39 mo	pre 2008		
10941971	34	1	2	2100	50.76	2	1	6	175	nr	nr	1	2	2	24 mo	pre 2008		
9932857	36	2	2	nr		nr	2	10	170	nr	nr	1	2	2	9 mo	pre 2008		
9880458	39	nr	1	3500	63.85	nr	2	6	95	nr	nr	1	2	2	24 mo	pre 2008		
9880458	38	nr	1	2600	7.45	nr	2	6	125	nr	nr	1	2	2	96 mo	pre 2008		
9880458	36	nr	1	3300	90.81	nr	2	6	150	nr	nr	1	2	2	84 mo	pre 2008		
9880458	39	nr	2	3160	45.90	nr	2	4	175	nr	nr	1	2	2	48 mo	pre 2008		
9880458	37	nr	2	2700	40.24	nr	2	4	100	nr	nr	1	2	1	36 mo	pre 2008		
9844114	38	1	1	3300	63.28	2	2	1	75	nr	nr	1	2	2	132 mo	pre 2008		
9822319	36	1	1	3090	81.64	2	2	1	100		1	1	2	2	3 mo	pre 2008		
9740207	AT	1	1	3900		2	2	14	178	nr	nr	1	2	1	18 mo	pre 2008		
9364305	36	1	2	2700	40.24	2	2	nr	175		1	1	2	2	3 mo	pre 2008		
9323563	33	1	1	2950	98.57	1	1	1	nr			1	2	1	7 mo	pre 2008		
8732907	AT	1	1	3350		2	2	7	50	1	nr	1	2	2	7 mo	pre 2008		
7959671	AT	1	2	4100		2	2	14	60	nr	nr	1	2	1	39 mo	pre 2008		
7959671	AT	1	1	4200		2	2	14	50	nr	nr	1	2	1	5 mo	pre 2008		
7959671	AT	1	1	3800		2	2	7	nr	nr	nr	1	2	2	58 mo	pre 2008		
8067796	38	1	1	3325	99.97	2	2	1	166	nr	1	1	2	1	9 d	pre 2008		
8067796	35	1	1	2880	84.01	2	2	2	200			1	2	1	4 mo	pre 2008		
8032396	35	1	2	2810	86.72	2	nr	3	120			1	2	2	7 mo	pre 2008		
1319670	37	nr	2	2700	40.24	2	2	4	200	nr	nr	1	2	1	37 mo	pre 2008		
1660676	AT	nr	1	3530		1	nr	1	150	nr	nr	1	2	2	72 mo	pre 2008		
1660676	37	nr	1	3300	79.66	1	nr	1	150	nr	nr	1	2	2	9 mo	pre 2008		
2759484	AT	1	2	2300		2	2	3	85	nr	nr	1	2	2	13 mo	pre 2008		
3977385	37	1	2	2500	22.04	2	2	2	nr	nr	nr	1	2	1	6 mo	pre 2008		
3977385	34	1	2	2200	60.96	2	2	4	nr			1	2	1	6 mo	pre 2008		
25635218	36	1	2	2800	70.98	2	2	1	120	1	1	1	2	1	9 mo	*MYO5B*	nr	nr
31559144	35	1	2	2445	61.37	2	2	1	nr	1	1	1	2	2	1 mo	*MYO5B*	Ile488Leufs*93	Ile488Leufs*93
30564347	36	2	1	2820		nr	nr	1	nr			1	2	1	3 mo	*MYO5B*	p.Arg900Serfs*4	N456S
30564347	37	2	1	3280	78.44	1	nr	1	nr	nr	nr	1	2	1	7 mo	*MYO5B*	p.K169G	p.K169G
30364420	35	1	1	3030	90.43	2	nr	5	nr	1	1	1	2	1	3 mo	*MYO5B*	nr	nr
29282386	AT	1	1	nr		2	nr	3	nr	nr	nr	1	2	2	1 mo	*STX3*		
28707991	AT	2	1	3300		2	2	2	nr	nr	nr	1	2	2	5 mo	nr		
25111220	36	nr	2	2950	99.86	2	nr	2	nr	1		1	2	2	36 mo	*MYO5B*	c2259-2262dup	c3163-3165dup
25111220	AT	nr	1	3290		2	2	7	nr	1	nr	1	2	2	24 mo	*MYO5B*	c1222A>T	c1582C>T
25111220	AT	nr	1	3570		1	nr	42	nr	1	nr	1	1	2	12 mo	*MYO5B*	c1222A>T	c1582C>T
25111220	AT	nr	1	3360		2	nr	5	nr	nr	nr	1	1	2	144 mo	no *MYO5B*		
25111220	40	nr	1	2380	0.07	2	1 (IUGR)	nr	nr	1	nr	1	2	2	156 mo	no *MYO5B*		
25111220	31	nr	1	1645	61.67	nr	nr	420	nr					2	288 mo	*MYO5B*	c656G>A	c4028T>C
23608388	36	nr	1	3355	92.46	2	2	1	nr	1	1	1	2	1	5 mo	*MYO5B*	p.Q149 * (f)	p.Q149 * (m)
22441677	AT	nr	1	nr		nr	nr	1	nr	nr	nr	1	2	2	60 mo	*MYO5B*	p.Gln1456 * (f)	p.Gln1456 * (m)
22152886	37	nr	1	nr		nr	nr	1	nr	1	nr	1	2	2	48 mo	nr		
22152886	37	nr	1	nr		nr	nr	3	nr	1	nr	1	2	2	48 mo	nr		
22030065	23	2	2	634		2	2	nr	nr					1	6 mo	*MYO5B*	p.Q149 *	p.Q341 *
21199752	36	1	1	3355	92.46	1	1	1	nr		1	1	2	1	6 mo	*MYO5B*	p.Q149 * (f)	p.Q341 * (m)
16454574	35	1	1	nr		2	2	1	nr			1	2	2	1.5 mo	pre 2008		
12028658	35	nr	2	2110	27.97	nr	nr	1	nr			1	2	2	4 o	pre 2008		
11208062	AT	1	2	nr		2	nr	nr	nr	nr	nr			2	36 mo	pre 2008		
10484813	36	1	1	2805	60.74	2	2	1	nr					1	37 mo	pre 2008		
10484813	37	1	1	3308	80.14	2	2	2	nr	nr	nr			2	132 mo	pre 2008		
10484813	35	1	2	2922	91.06	2	2	2	nr					1	7 mo	pre 2008		
10484813	34	1	1	2558	80.48	2	2	2	nr					1	4 mo	pre 2008		
9364305	39	1	1	3000	19.22	2		3	nr	1	nr	1	2	2	8 mo	pre 2008		
9142307	AT	1	1	3570		2	2	2	nr	nr	nr			1	29 mo	pre 2008		
2891946	33	nr	2	1900	53.70	nr		1	nr					1	11 mo	pre 2008		
2891946	39	nr	2	nr		nr		1	nr	nr	nr			1	22 mo	pre 2008		
28899465	39	1	2	2850	18.15	nr		2	nr	nr	nr	1	2	2	6 mo	*MYO5B*	p.R656C	c.310+2Tdup
8119548	AT	nr	2	2870		2		nr	nr	nr	nr			2	53 mo	pre 2008		
25635218	36	1	2	2800	70.89	2	2	3	nr	1	1	1	2	1	9 mo	*MYO5B*	nr	nr
33525641	36	nr	2	nr		nr	nr	nr	nr	1	nr	1	nr	1	1.5 mo	*MYO5B*	p.I416N	p.I416N
33525641	PT	nr	2	nr		nr	nr	nr	nr	2	nr	1	nr	1	7 mo	*MYO5B*	p.R656C	p.K672 *
33525641	34	nr	1	nr		nr	nr	nr	nr	1	nr	1	nr	1	5 mo	*MYO5B*	p.D492G	p.D492G
33525641	35	nr	2	nr		nr	nr	nr	nr	2	nr	1	nr	2	11 mo	*MYO5B*	p.K246 *	p.V871Gfs * 33
33525641	33	nr	1	nr		nr	nr	nr	nr	1	nr	1	nr	1	6 mo	*MYO5B*	c.1323-2A > G	c.1323-2A > G
33525641	35	nr	2	nr		nr	nr	nr	nr	1	nr	1	nr	1	7 mo	*MYO5B*	p.I497F	p.E574 *
33525641	36	nr	1	nr		nr	nr	nr	nr	2	nr	1	nr	1	5 mo	*MYO5B*	p.K672 *	p.K672 *
33525641	31	nr	1	nr		nr	nr	nr	nr	1	nr	1	nr	2	120 mo	*MYO5B*	c.838 + 1G > A	p.H1580Qfs * 1
33525641	36	nr	2	nr		nr	nr	nr	nr	1	nr	1	nr	1	36 mo	*MYO5B*	p.D570Gfs * 5	p.G336R
33843415	34	1	1	nr		nr	2	2	200	nr	1	1	2	1	1 mo	nr		
30909251	FT	2	1	nr		1	1	1	nr	nr	nr	1	1	2	15 mo	*STX3*		
**NON-MVID PFIC-6 CASES**																		
28027573	AT		1							1						*MYO5B*	c.3538-1G>A (f)	c.2414+5G>T (m)
28027573	AT		1							1						*MYO5B*	p.R401C (f)	p.Q341X (m)
28027573	AT		2							1						*MYO5B*	p.Q1079H (f)	p.S535N (m)
28027573	AT		1							1						*MYO5B*	p.C266R	p.C266R
28027573	AT		1							**1**						*MYO5B*	p.S583N	p.I934S
28027573	AT		1							1						*MYO5B*	p.R697Gfs*74	c.4852+11A>G
28027573	AT		1							1						*MYO5B*	p.R1016X (m)	p.S158F (f)
28027573	AT		2							1						*MYO5B*	p.R824C	p.R824C
28027573	AT		1							1						*MYO5B*	p.R824C (m)	mono-allelic
28027573	AT		1							1						*MYO5B*	p.R379P (f)	mono-allelic
27532546	41	1	2							1						*MYO5B*	p.Arg92Cys	p.Arg799Trp
27532546	39	1	1	3220						1						*MYO5B*	p.Tyr119Cys	p.Tyr119Cys
27532546	AT		1							1						*MYO5B*	p.Arg379Cys	Phe636Leufs*2
27532546	AT		1							1						*MYO5B*	c.1753-1G>T	p.Arg799Trp

Legend

(shaded box)	: preterm births (see sheet 2)
(white box)	: term births (see sheet 3)
nr	: not reported
AT, PT, FT	: at term, preterm, full term
